# Tuning Neural Synchronization: The Role of Variable Oscillation Frequencies in Neural Circuits

**DOI:** 10.3389/fnsys.2022.908665

**Published:** 2022-07-08

**Authors:** Eric Lowet, Peter De Weerd, Mark J. Roberts, Avgis Hadjipapas

**Affiliations:** ^1^Department of Biomedical Engineering, Boston University, Boston, MA, United States; ^2^Department of Cognitive Neuroscience, Faculty of Psychology and Neuroscience, Maastricht University, Maastricht, Netherlands; ^3^Medical School, University of Nicosia, Nicosia, Cyprus; ^4^Center of Neuroscience and Integrative Brain Research (CENIBRE), University of Nicosia, Nicosia, Cyprus

**Keywords:** synchronization, frequency, Arnold tongue, neural circuits, oscillation

## Abstract

Brain oscillations emerge during sensory and cognitive processes and have been classified into different frequency bands. Yet, even within the same frequency band and between nearby brain locations, the exact frequencies of brain oscillations can differ. These frequency differences (detuning) have been largely ignored and play little role in current functional theories of brain oscillations. This contrasts with the crucial role that detuning plays in synchronization theory, as originally derived in physical systems. Here, we propose that detuning is equally important to understand synchronization in biological systems. Detuning is a critical control parameter in synchronization, which is not only important in shaping phase-locking, but also in establishing preferred phase relations between oscillators. We review recent evidence that frequency differences between brain locations are ubiquitous and essential in shaping temporal neural coordination. With the rise of powerful experimental techniques to probe brain oscillations, the contributions of exact frequency and detuning across neural circuits will become increasingly clear and will play a key part in developing a new understanding of the role of oscillations in brain function.

## Main Text

In the mammalian brain, oscillations and synchronization have been found ubiquitously at the level of single neurons, local neural circuits and brain-wide networks, from deep brain nuclei to neocortex and across a variety of species (Buzsáki et al., 2013). The association of oscillatory synchronization with sensory-motor and cognitive processes during sleeping and waking has been well documented. Further, various symptoms are associated with abnormal patterns of synchronization in psychiatric and neurological conditions including epilepsy (da Silva et al., 2003), Parkinson’s disease (Little and Brown, 2014; McGregor and Nelson, 2019) and schizophrenia (Haig et al., 2000; Herrmann and Demiralp, 2005). Unraveling the functions of neuronal oscillatory synchronization has thus been one of the prime objectives of systems neuroscience.

It is common to classify rhythms into broad frequency bands including the delta (2–4 Hz), theta (4–8 Hz), alpha (8–12 Hz), beta (14–25 Hz), and gamma (30–100 Hz) bands. This broad frequency classification has been fruitful in correlating amplitude modulations within specific frequency bands with specific brain regions and/or specific task variables. However, this classification has not enabled a mechanistic interpretation of the range of oscillation frequencies contained within these broad frequency bands, thereby preventing a full understanding of the neural networks that generate the oscillations. Here, we summarize accumulating experimental evidence showing that oscillations within the same broad frequency band may express systematically different frequencies across brain regions and that precise oscillation frequencies are systematically modulated as a function of sensory, motor and cognitive variables. Referring to concepts from synchronization theory (Kopell and Ermentrout, 2002; Pikovsky et al., 2002; Izhikevich, 2007), we then discuss the significance of such systematic tuning of the oscillation frequency for understanding rhythmic coordination and communication in the brain.

### The Preferred Oscillation Frequency Is Diverse Across and Within Brain Regions

Although frequency bands have to some extent become associated with specific regions and functions (e.g., gamma with visual processes in low to intermediate visual cortex, and theta with memory processes in hippocampus and frontal lobe), activity in each frequency band can be found in large networks throughout the brain (see Table 1). For example, gamma-band rhythms during visual processing can be found over various visual, parietal and frontal cortical areas (Fries, 2009; Gregoriou et al., 2009; Bastos et al., 2014) as well as subcortical areas (Colgin et al., 2009; Wulff et al., 2009). Another example are theta rhythms which can be co-expressed in different subcortical structures like the hippocampus as well as in cortical regions (Lakatos et al., 2005). These rhythms not only co-occur under similar experimental conditions, but they exhibit marked temporal coordination both within and across brain areas, which changes as a function of sensory stimulus properties, cognition states and animal behavior (Fries, 2005, 2015).

**TABLE 1:** A - not exhaustive – overview of observed frequency shifts and differences (detuning) for classically defined frequency bands (delta, theta, alpha, beta, gamma) in the central nervous system.

Frequency-bands	Brain structures	Experimentally reported frequency shifts/differences
Gamma (∼30–100Hz)	Neocortex Hippocampus	(Traub et al., 1996; Gregoriou et al., 2009; Lima et al., 2010; Ray and Maunsell, 2010; Ahmed and Mehta, 2012; Bosman et al., 2012; Jia et al., 2013; Roberts et al., 2013; van Pelt and Fries, 2013; Lowet et al., 2017)
Beta (∼15–35Hz)	Neocortex Basal ganglia	(Salmelin and Hari, 1994; Neuper and Pfurtscheller, 2001; Kilavik et al., 2012; Lundqvist et al., 2016; Canessa et al., 2020; Moënne-Loccoz et al., 2020)
Alpha (∼8–12Hz)	Neocortex Thalamus	(Haegens et al., 2014; Zhang et al., 2018; Green et al., 2022; Sato, 2022)
Theta (∼4–10Hz)	Neocortex Hippocampus	(Giocomo et al., 2007; Goutagny et al., 2009; Axmacher et al., 2010; Patel et al., 2012; Zhang and Jacobs, 2015; Zhang et al., 2018)
Delta (∼1–4Hz)	Spinal cord	(Kopell and Ermentrout, 1986, 1988; Cohen et al., 1992; Grillner et al., 1995)

*It includes frequency shifts with sensory, cognitive or motor variables and frequency differences observed across structures.*

Intuitively, if oscillatory cycles in one brain region are to coordinate with cycles of another brain region, one assumes that they must share a common frequency. Consistent with this prevalent assumption is the wide use of (spectral) coherence to quantify interactions (Lowet et al., 2016). Coherence however, assumes stationarity of the underlying oscillations (for instance a stable frequency) and linearity of the relationship between them. Any cross-frequency interactions, even across very small frequency differences, are therefore not accounted for in measures of coherence. However, experimental observations showing that neural rhythms across and within brain regions often have somewhat different frequencies show that the stationarity and linearity assumptions required for the calculation of coherence are often violated. For example, small but systematic differences in visual cortical gamma-band rhythms are well documented in the primate visual system. Using a high-density ECoG grid spanning various visual cortical areas in the macaque monkey, Bosman et al. (2012) reported that the preferred gamma frequency was systematically higher (by a few Hz) in V1 than in V4. Different gamma-band frequencies have been also reported between V1 and V2 (Lowet et al., 2018).

Beyond the small differences in gamma frequencies across cortical areas, small differences in preferred frequency can also occur locally within the same cortical area. A gamma-band frequency gradient as a function of eccentricity (fovea to periphery) in V1 has been established (Lima et al., 2010; van Pelt and Fries, 2013; Lowet et al., 2017). In addition, Zhang, (Zhang et al., 2018) has recently observed systematic theta and alpha waves traveling over cortex as recorded by a high-density ECoG array. They found that the direction of the traveling waves was systematically related to frequency gradients over cortical space. Similarly, theta rhythmic resonance in the rodent hippocampal-entorhinal system has also been shown to exhibit specific frequency changes over space (Giocomo et al., 2007; Shay et al., 2012). These different frequency gradients have been hypothesized to be the main cause of observed theta traveling waves occurring across the hippocampus (Zhang and Jacobs, 2015), which has received further experimental support from *in vitro* recordings (Goutagny et al., 2009).

### The Precise Frequency Changes With Sensory and Cognitive Variables

Here, we will provide evidence that oscillation frequency not only differs as a function of anatomical location, but also depends systematically on the animal’s sensation, cognition and behavior (see Table 1).

Gamma-band synchronization is a prime example of oscillations exhibiting marked variability in oscillation frequency, due to a high sensitivity to sensory and cognitive conditions. Various computational studies have shown that the frequency control of the pyramidal-interneuron gamma (PING) mechanism is highly sensitive to the excitation level of excitatory and inhibitory neurons within the PING circuit (Brunel and Wang, 2003; Tiesinga and Sejnowski, 2009; Zachariou et al., 2015), in line with experimental observations showing that increases in gamma frequency are accompanied by increases in firing rate (Ray and Maunsell, 2010; Jia et al., 2013; Roberts et al., 2013). Hence, sensory, cognitive and motor parameters that change the excitability-inhibition balance (Brunel and Wang, 2003; Buzsáki and Wang, 2012) of coordinating neurons will likely also change the oscillation gamma frequency. The dependence of the oscillation frequency on network state is thus expected to be a general property of brain rhythms (Brunel and Wang, 2003; Wang, 2010).

**FIGURE 1:**
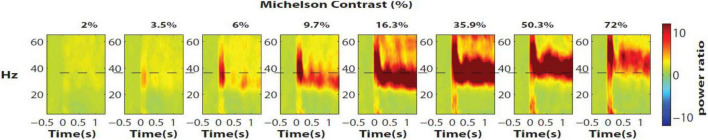
Gamma frequency shifts in macaque V1 as a function of the basic visual input parameter contrast (single electrode contact example from data published in Roberts et al., 2013 & Hadjipapas et al., 2015). Gamma peak frequency shifts to higher frequencies with visual contrast. The monkey maintained fixation while stimuli were presented away from fixation for > 1 s. Zero on the X-axis represents the presentation onset of the grating stimulus, which remained in the neuronal RFs for the remainder of the trial.

The relation between gamma-band frequency and sensory variables has been increasingly studied over the last decade in primate and human early visual cortex. Gamma-band frequency decreases with increasing stimulus size (Gieselmann and Thiele, 2008; Ray and Maunsell, 2010; Veit et al., 2017), which has been linked to increased recruitment of horizontal inhibition. Striking increases in gamma frequency in V1 as well as V2 have been observed with visual stimulus contrast (Figure 1) with reported shifts of about ∼20 Hz in monkey V1 and ∼10 Hz in human V1 (Roberts et al., 2013; Hadjipapas et al., 2015). Gamma frequency is also modulated as a function of moving speed (Friedman-Hill et al., 2000; Swettenham et al., 2009; Jia et al., 2013), motion direction (Feng et al., 2010), color (Peter et al., 2019) and stimulus complexity (Brunet et al., 2013; Hermes et al., 2015). Importantly, not only is the frequency shift dependent on global stimulus properties, but neuronal groups in nearby locations within area V1 or V2 may express different frequencies depending on the precise stimulus properties in these neural groups’ receptive fields (Gieselmann and Thiele, 2008; Ray and Maunsell, 2010; Lowet et al., 2017, 2018). This indicates that the generative mechanisms of gamma are highly localized. For example, local image contrast can induce distinct gamma frequencies in visual cortical locations with adjacent receptive fields (Ray and Maunsell, 2010; Lowet et al., 2017). In line with these findings, optogenetically increasing the excitability of pyramidal neurons in cat V1 (Area 17) leads to an increase in the gamma frequency (Ni et al., 2016; Lewis et al., 2021). These findings suggest a generic mechanism of how excitatory drive changes the gamma frequency; confirming prior biophysical modeling studies (Traub et al., 1996; Tiesinga and Sejnowski, 2009, 2010).

Recent studies have shown that cognitive processes also systematically influence the frequency of rhythms. Visual spatial attention (Bosman et al., 2012) has been observed to shift the V1 gamma band to higher frequencies when the monkey attended the stimulus overlaying the corresponding receptive field, whereas simultaneously recorded unattended V1 locations had a lower frequency. Similar frequency shifts by spatial attention have also been observed in V4 and FEF (Gregoriou et al., 2009). Gamma peak frequency in human MEG has been found to also be predictive of performance in an orientation discrimination task across individuals (Edden et al., 2009). The dependence of the precise oscillation frequency on behavior has also been reported for other frequency bands. For example, the systematic increase of hippocampal theta frequency with locomotion speed (McFarland et al., 1975; Bender et al., 2015; Goyal et al., 2020) has been well documented. Further, the capacity of working memory has been directly linked to the frequency of theta rhythms. Axmacher et al. (2010) showed that subjects with higher working memory load displayed a decrease in their hippocampal theta frequency, in line with a model in which longer theta cycles permit representations of more items (Axmacher et al., 2010). Consistent with this, artificially reducing the theta frequency over parietal cortex using transcranial magnetic stimulation increased the working memory capacity (Wolinski et al., 2018). Another study showed that alpha peak frequency predicts working memory capacity across age span (Clark et al., 2004). These and other studies demonstrate that oscillation frequency is a variable parameter that interacts with basic neural network properties to represent changes in perception and cognition.

The mechanism of frequency regulation, although not largely understood, is likely to depend on the oscillation generation mechanisms, which involve intracellular properties (i.e., bursting) of various cell types, network properties (i.e., synaptic strengths and time constants, the nature of input to the network) as well as involving different neuron transmitters and neuromodulators (i.e., acetylcholine). The generation mechanisms of gamma oscillations have been studied extensively over the last decades using excitatory-inhibitory networks models (PING or ING models), which can reproduce many features of experimentally observed gamma oscillations including shifts of the gamma frequency and the gamma oscillation amplitude (Brunel and Wang, 2003; Tiesinga and Sejnowski, 2010; Roberts et al., 2013; Lowet et al., 2015). In primate visual cortex, increasing visual contrast, a proxy of feedforward excitatory drive (Sclar et al., 1990), is known to increase the frequency of gamma oscillations (Ray and Maunsell, 2010; Jia et al., 2013; Roberts et al., 2013; Hadjipapas et al., 2015; Self et al., 2016). This can be captured well in PING networks, in which excitatory units receives increasing amount of excitatory drive (Traub et al., 1996; Tiesinga and Sejnowski, 2009; Roberts et al., 2013; Lowet et al., 2015; Zachariou et al., 2021). However, the precise change in the frequency depends also on the drive to inhibitory neurons and other network parameters, which often remain unknown (Zachariou et al., 2021). It has also been observed that visual attention can increase the frequency of visual cortical gamma oscillations (Bosman et al., 2012). While this might result from a change in excitatory drive to pyramidal and inhibitory neurons, similar to what occurs for increased contrast, it is likely that the biophysical mechanisms differ. For example, top-down or feedback axonal projections might target different parts of the dendritic tree thus engaging different intracellular dynamics, and might preferentially activate different interneurons and act on different spatial and temporal scales. Further, the role of neuromodulators like acetylcholine might play an important role in behavioral state-dependent changes in oscillation properties, as shown for example in the role of cholinergic modulation of hippocampal theta frequency (Heys et al., 2010; Newman et al., 2013). In addition, it is of note is that differences in the neural mechanisms underlying frequency change can lead to different network effects, which can have implications for the functional significance of the frequency change. For example, enhanced activity of feedback inhibition might reduce the oscillation frequency but increase synchronization (oscillation amplitude) in PING networks. In contrast, while reducing excitatory drive to pyramidal neurons also reduces oscillation frequency, it decreases synchronization.

In summary, various sensory and cognitive factors appear to modulate detuning and may do so by one or most likely several potential mechanisms. One cannot safely conclude from an observed change in detuning which of the potential underlying mechanisms is operating without additional information about the network. Therefore, although experimental manipulations of detuning offer an important route toward understanding the neuronal effect of the manipulation, this understanding requires information about network properties and an assessment of network effects.

### Frequency Is Critical for Spatial Synchronization and Phase Relations in Oscillator Networks

Synchronization is often conceptualized as the maintenance of a stable (or at least bounded) phase difference (relation) between two oscillators (Pikovsky et al., 2002). It is intuitive that, in the absence of noise, a pair of frequency-stable oscillations can have a stable phase-relation if their frequencies match. For this to happen, no interaction *per se* is needed since in the absence of noise the phase-relation will remain constant over time. Alternatively, if a pair of oscillators do not interact and they have a frequency difference, their phase-relation will change continuously with a rate determined by their frequency difference (detuning), a phenomenon termed *phase precession*. Synchronization as a process, as formulized in the framework of weakly coupled oscillators (Kopell and Ermentrout, 1986, 2002; Strogatz and Stewart, 1993; Izhikevich, 2007), only comes into play when oscillations of different and variable frequencies (detuned oscillators) in different neural groups (oscillators) coordinate around a preferred phase-relation in order to enable neural communication. To achieve this, the phase precession due to the detuning needs to be counteracted through interaction (coupling), which is defined by the so called phase response curve [PRC, for review (Schwemmer and Lewis, 2012)]. Oscillatory interactions lead to phase advances or delays (described by the PRC), which translate to systematic changes of the oscillators’ frequencies that ultimately reduces phase precession. Synchronization will make a slower oscillator speed up when entrained by a faster oscillator and vs. Hence, synchronization essentially involves continuous phase adjustments (deceleration or acceleration) in order to counter-act detuning and thereby balances the oscillations around a preferred phase-relation. This short overview of synchronization highlights that synchronization is a highly non-stationary and non-linear process (Kopell and Ermentrout, 2002; Pikovsky et al., 2002; Izhikevich, 2007) and that frequency detuning has a key role in the process.

#### Oscillation Frequencies: Natural Frequency and Detuning

In simple physical systems, such as a pendulum, the preferred frequency of an object to oscillate or resonate can be a relative stable property. For example, the pendulum has a characteristic frequency that depends on fixed factors, such as the length of the suspension rope. As we have discussed above, the frequency of neural oscillations can differ between brain areas and can be variable over time depending on sensory, cognitive and motor variables that modulate the underlying neural oscillation generation mechanism.

**FIGURE 2:**
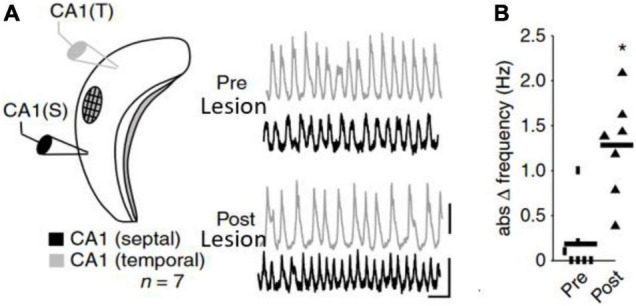
**(A,B)** *In vitro* recording from mouse CA1 from Goutagny et al. (2009). **(A)** Two electrodes in septal and temporal part of CA1 recorded the LFP characterized by theta rhythms. The septal and temporal theta rhythms are synchronized. After lesioning the CA1 in between septal and temporal pole of CA1, the theta rhythms became independent and **(B)** started to oscillate at different frequencies revealing the underlying frequency difference (detuning). Figures adapted from Goutagny et al. (2009).

The frequency of an un-perturbed neural oscillator is called its natural (eigen-, initial or intrinsic) frequency. In practice an oscillator’s natural frequency and its detuning with respect to other oscillators is difficult to measure in a neuroscientific context because the ‘oscillators’ in the living, behaving animal brain are always coupled. In order to observe the natural/intrinsic frequency of an oscillator, it must be decoupled and unperturbed. Here we use two examples to illustrate this. The first illustration pertains to studies of the circadian rhythm: It was observed that the circadian rhythm follows its natural frequency when subjects were decoupled from the day-night rhythm (Strogatz and Stewart, 1993). Thus, without the extrinsic influence of the days turning to nights on a 24-h cycle participants’ sleep/wake and other circadian rhythms were revealed to have an intrinsic frequency of around 25 h. The second example (Figure 2) was reported by Goutagny et al. (2009) in a natural *in vitro* hippocampal CA1 preparation. Theta rhythms in CA1 between septal and temporal regions were synchronized with nearly matching frequencies. Yet, when an intermediate region between the two recording sites was lesioned, thereby disrupting connectivity, the two CA1 regions started to oscillate at distinct preferred frequencies. Thus, while the observed/expressed frequencies in the original preparation were matched, the natural frequencies were not. The matched frequencies were the result of synchronization taking place.

As shown above, the natural frequency of brain oscillations and hence detuning can change systematically due to sensory, motor and cognitive variables at short time scales. In noiseless oscillatory systems, detuning is the main obstacle that synchronization has to overcome to achieve phase coordination. However, in biological systems there are additional obstacles as oscillations are commonly highly variable over time and noisy. Apparent random frequency variability is often called phase noise and it limits the ability of oscillators to remain stable at their preferred phase relation. Notably, frequency variability over time can reflect in part the expected features of interacting oscillators, and this aspect of frequency variability is a feature of synchronization, rather than an obstacle. However, phase noise (and hence frequency variability) in addition of randomness may also include other unaccounted systematic (deterministic) complexities like inherent short-term instability of oscillation generation mechanism (e.g., chaotic rhythms) or complexities of the underlying network (due to cross-frequency coupling, or other interactions among multiple rhythms).

#### The Arnold Tongue(s): The Structure of Synchronization

**FIGURE 3:**
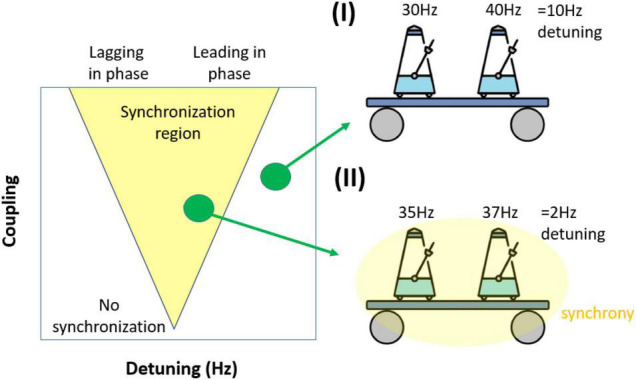
Illustration of how frequency differences (detuning) impact synchronization. (I) Coupled oscillators (metronomes) are not synchronized due to large differences in their preferred frequency. (II) Coupled oscillators are synchronized as they can overcome their smaller frequency difference (detuning). The simplified synchronization region mapped as a function of frequency difference (detuning) and coupling. The triangular synchronization region is called the Arnold tongue. Notably, although the synchronization region will generally be triangular, it will not necessarily be symmetrical, depending on the underlying dynamical system, the connectivity pattern and time delays.

In an uncoupled system, frequency differences between two oscillators lead to phase precession. Larger frequency differences (detuning) produce faster phase precession. However, in a dynamic system of coupled oscillators, synchronization counteracts phase precession. Synchronization requires moment-to-moment changes of the oscillation frequency. This change of frequency over time is what, from a dynamic perspective, is called interaction. The stronger a pair of oscillators interact, the stronger the frequency modulation becomes, which in turn corresponds to phase advances or delays. This fundamental interplay between the amount of detuning and the amount of interaction is what is reflected in the so-called Arnold tongue (Figure 3). The Arnold tongue describes the regions of synchronization characterized by high phase coordination (phase-locking) in the 2-dimensional space of detuning (drives phase precession) and coupling strength (drives interaction). The region looks like an inverted triangle (hence the term ‘tongue’). For the purpose of this review, we will only consider the synchronization region of 1:1 phase-locking and ignore higher order phase-locking regimes [1:2, 1:3,…, (Pikovsky et al., 2002)].

The inverted triangular shape of the Arnold Tongue (Figure 3) shows that to achieve synchronization, the increased phase precession at higher levels of detuning needs to be counter-balanced by increasing coupling. An understanding of how these two forces compete to form the Arnold tongue leads to two important insights. First, if interaction is strong enough to fully counterbalance the detuning, the resultant oscillations frequencies will match. In turn this means that experimentally-measured matched frequencies across brain regions does not necessarily mean that these rhythms also had matching natural frequencies, as was demonstrated by Goutagny et al. (2009) in the *in vitro* hippocampus recordings (see Figure 2). Second, if interaction is strong enough to counterbalance the detuning, the detuning is converted into a phase difference. Usually, the oscillation with the higher natural frequency will lead the oscillation with the lower natural frequency. These phase differences can emerge without any time-delay in the interaction. Hence, an experimentally measured phase difference can emerge due to synchronization and does not necessarily imply a time-delay in the underlying connectivity. In practice, time delay and detuning will contribute to the observed phase difference in rhythmic neural interactions.

It is important to emphasize that the strongest phase-locking does not necessarily occur at zero detuning, but depends on how the interaction influences the rhythms’ frequencies. The interaction is defined by the underlying phase response curves (PRCs). For example, if synaptic input leads to an acceleration of the down-stream neurons, then it is preferable that the up-stream neurons have a higher frequency compared to the down-stream neurons (Arnold tongue is one-sided). Alternatively, if interaction only leads to deceleration (e.g., inhibition delaying spiking), then the up-stream neurons should have a slower frequency compared to the down-stream neurons. When the interaction has both accelerating and decelerating components (also known as type II PRC compared to type I PRC, [Ermentrout, 1996; Ko and Ermentrout, 2009; Schwemmer and Lewis, 2012)], the detuning between the oscillators will synchronize with positive or negative. The PRC is not a fixed property for a given neural oscillation, but it can change with changes in the underlying intracellular or network properties. A prime example is the well-documented change from mono-phasic type I PRC (either negative or positive) to bi-phasic type II PRC (positive and negative) with cholinergic tone (Stiefel et al., 2008, 2009), which will have dramatic effects on the shape of the Arnold tongue.

According to the theory of weakly coupled oscillators, the amount of detuning that will modulate phase-locking and shift the preferred phase relation depends on the PRC of the oscillations and the coupling strengths between the oscillations. Notably, the amount of detuning that will modulate phase-locking and shift the preferred phase relation does not depend necessarily on the frequency-band of the oscillation; for example, the effect of 1 Hz detuning might be similar whether the oscillation is in the theta range (4–10 Hz) or in the gamma range (30–100 Hz). However, little is known at present about how the PRCs and coupling strength differ between the different brain oscillation bands. Further, it remains unclear whether the amount of frequency variability and phase noise might differ depending on the time scale of brain oscillations.

**FIGURE 4:**
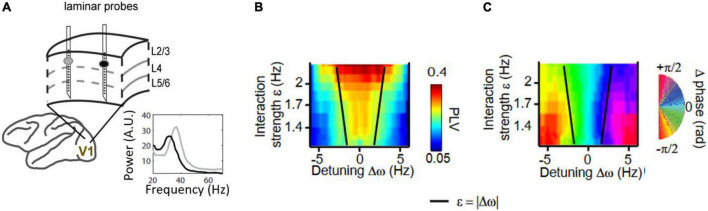
**(A)** Gamma-band synchronization studied between nearby cortical V1 locations in behaving macaques. Two or three laminar probes to record current source densities (CSD) were inserted in V1 while gratings of spatially variable contrast were shown. Visual contrast is known to modulate the frequency of V1 gamma rhythms. **(B)** The gamma-band phase-locking value (PLV) was mapped as a function of frequency difference and interaction strength (estimated by cortical distance). The gamma PLV between sites exhibited an Arnold tongue structure. **(C)** Same as **(B)** but mapping the preferred phase relation in the gamma range between V1 sites. Preferred phase is defined here as the mean phase difference between two gamma rhythmic V1 signals. Figures adapted from Lowet et al. (2017).

In spite of the important insights that can be garnered from direct experimental mappings of the Arnold tongue in neural rhythms, studies that do so are still scarce. Yet, two recent studies have been able to map the Arnold tongues experimentally. Notbohm et al. (2016) used flickering visual stimuli at different frequencies and intensities while measuring alpha-range (8–12 Hz) evoked responses in humans. They found a synchronization region between the external oscillatory force provided by the visual stimulus flickering frequency and visual cortical alpha with a shape as expected from an Arnold tongue. Lowet et al. (2017) systematically mapped the synchronization region of simultaneous recorded gamma rhythms from different locations in awake macaque V1 and V2 while changing gamma frequency naturally using local image contrast (Figure 4). An inverted triangular-shaped synchronization region was observed that determined not only the observed phase-locking between gamma-synchronized cortical regions, but also their preferred phase relation.

### The Neurobiological Functions of Oscillation Frequency Diversity

We have described how frequency diversity of interacting neural rhythms is present across the brain and is modulated by sensation, cognition and behavior. We have also outlined synchronization theory and highlighted that frequency differences among rhythms have important consequences for how they synchronize and what phase-relation they will prefer. In the present section we will discuss more broadly the implications of oscillation frequency shifts for brain function.

#### Frequency Gradients for Limiting Amount of Neural Synchrony

Flexible regulation and limitation of synchronization in cortical and subcortical brain areas is of critical importance for healthy neural functioning. Abnormal levels of synchronization dynamics of neural activity can have severe negative impact as exemplified for beta rhythms in Parkinson’s disease (Little and Brown, 2014). Frequency diversity in the brain can be viewed as a kind of safety mechanism, which acts to prevent abnormal level of synchronization, and to permit healthy brain function. It is well established that frequency diversity in a network of coupled oscillators (Kuramoto model) reduces global synchronization and shapes clustering (Ermentrout and Kopell, 1984; Chawanya et al., 1993; Strogatz and Stewart, 1993). We have mentioned that there is experimental evidence of systematic frequency gradients in cortical and subcortical areas across different oscillation bands. For example, the preferred gamma frequency decreases as a function of eccentricity (fovea to periphery) in macaque area V1 (Lima et al., 2010). Theta frequency preference in entorhinal cortex and hippocampus show systematic spatial gradients (Shay et al., 2012). In primary sensorimotor cortex the representations of the hand and foot areas exhibit distinct frequencies of beta event related synchronization within the broader beta band (Pfurtscheller et al., 2000; Neuper and Pfurtscheller, 2001). These observations can be seen as supporting the conjecture that slight (but sufficiently large) frequency differences in different network parts are functionally beneficial in the maintenance of related but separate cognitive processes.

**FIGURE 5:**
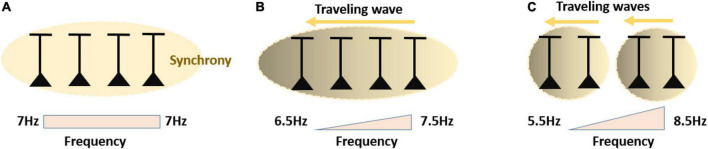
**(A)** Neurons with matching preferred oscillation frequencies will have a strong tendency to synchronize into one oscillating assembly spiking at same preferred phase. **(B)** Neurons characterized by a fine frequency gradient will still likely synchronize into one oscillating assembly, yet neurons with slightly higher preferred frequency will spike at an earlier phase. Spatially, this leads to a traveling wave. **(C)** Neurons characterized by a large frequency gradient, will likely synchronize in distinct sets/clusters of synchronized assemblies oscillating at different frequencies along the gradient. Within the synchronized assemblies, the gradient is converted into phase differences corresponding to local traveling waves.

Synchronization theory predicts that spatial frequency gradients will limit the extent of neural synchronization, depending on the slope of the frequency gradient and how strongly neurons interact (Figure 5). This is an interplay well represented by the Arnold tongue. Change in frequency preference may be due to differences in intracellular kinetics, different balance of cell types and connectivity properties. While frequency gradients may be implemented through different mechanisms, they might have the same dynamical function: to keep synchronization sufficiently locally and in a healthy regime. For instance, it may be functionally advantageous that when the hand area is activated, that no strong activity is actually propagated through the relatively dense anatomical connections to the foot area. Thus, the limited domain in synchronization serves a representational role by segregating the stimulated area from nearby areas that it is coupled to. Similar considerations apply to the foveal versus peripheral representations in the gamma band mentioned previously. Thus, the targeted absence of synchronization because of large intrinsic frequency differences that cannot be reconciled by the coupling may be utilized functionally.

#### Frequency Gradients Organize Traveling Waves and Information Flow Within and Across Brain Areas

Synchronization theory predicts that when oscillations synchronize, even if not completely, their initial frequency differences will be converted into a preferred phase relation. For example, in many cases the oscillation with the higher natural frequency will lead in phase. This means that neurons involved in the higher frequency oscillation will fire earlier than neurons involved in the lower frequency oscillation. Applied to a frequency gradient, this results into a traveling wave which spreads down the frequency slope (Ermentrout and Kopell, 1984; Ermentrout and Kleinfeld, 2001). The speed of the traveling waves relates to the slope of the frequency gradient. The existence of traveling waves along human cortex has recently been demonstrated (Zhang et al., 2018). Related work has shown that neural rhythms can be coordinated across different cortical areas in a directed manner. For example, primate cortical gamma rhythmic coordination seems to be organized in a feedforward manner from primary to higher-order visual and associative areas (Bastos et al., 2014). In addition, it has been observed that V1 gamma rhythms have a higher frequency than V4 gamma rhythms (Bosman et al., 2012). This opens the interesting possibility that separate streams of information across cortical hierarchies may be coordinated by specific rhythms with the direction guided by the frequency gradient. Notably, given the fact that hardwired biases in the local frequency can be flexibly influenced by cognitive states or sensory input, a cortical location may switch from follower” to leader” by flipping the frequency gradient. This possibility has been confirmed by Lowet et al. (2017), who showed that a location in V1 displaying a higher gamma frequency due to high-contrast stimulation acted as a leader” compared to a neighboring V1 location displaying a lower frequency due to low-contrast stimulation. More studies are required to test whether by shifting the rhythm’s frequency experimentally, a cortical location can be switched from a follower” to a leader” relative to another cortical area. From a developmental perspective, an inherent frequency gradient from primary to higher-order area might support the development of direct and asymmetric anatomical projections through spike-timing dependent plasticity during maturation (Caporale and Dan, 2008; Lee et al., 2009; Markram et al., 2012).

#### Flexible Regulation of Synchronization for Sensation and Cognition

The diversity of oscillation frequencies across the brain is not a static property, but is shaped by sensory input, cognition and behavior. Influential theories of how neural synchronization contribute to brain function require that synchronization emerges in a controlled and flexible manner in time and space. Temporal coordination of spike timing has consequences for information coding, signal transmission and plasticity. Whereas neural communication determined by anatomical connectivity is relatively fixed over time, neural synchronization can increase the repertoire of coordination patterns, change flexibly according to behavior and may eventually modify the anatomical connectivity based on experience. This flexibility of synchronization to coordinate neural activity has motivated theories highlighting synchronization as mechanism for sensory segmentation and grouping [Binding by synchrony,” (Singer and Gray, 1995; Eckhorn et al., 2001)] or to selectively route information across cortical areas as needed for visual attention (Fries, 2005, 2009; Akam and Kullmann, 2014).

**FIGURE 6:**
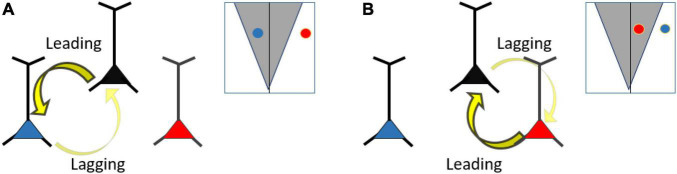
**(A)** Frequency differences (detuning) regulate information flow. Here, bi-directional connectivity and symmetrical Arnold tongue is assumed. Reducing the detuning between two neurons, such that they fall within the Arnold tongue (the black and blue neuron), will lead to stronger phase entrainment compared to a neuron pair with larger detuning (black and red neuron). The neuron with the higher frequency (black neuron) will lead in phase relative to the slower neuron (blue neuron). **(B)** Same as **(A)**, but changing the frequency relationship between the blue, black and red neuron. Here, the black and red neurons have a smaller detuning than the black and blue neuron. The red neuron has a higher frequency than the black neuron and thus leading in phase.

The appeal of these proposals is that synchronization does not need to follow anatomical connectivity *per se*, but is able to shape effective” connectivity (Fries, 2009). However, how changes in effective connectivity are implemented in the brain remains largely unknown. From the viewpoint of synchronization theory (and thus the Arnold tongue), there are two main ways: The first is an increase in oscillation *amplitude*, which ensues from synchronized spikes and would result in a more effective activation of connected down-stream neurons (Fries et al., 2001a). This corresponds to an enhancement of the coupling. Dynamically, an increase in interaction strength means the ability to more strongly modulate the frequency of the receiving rhythm and thereby to influence phase precession. The second way to shape effective connectivity is to modulate the *detuning* (Figures 6A,B). This facilitates rhythms to reach, maintain or regain their preferred phase relation. Due to the reduced detuning, the rhythms will reach stronger synchronization with the same degree of interaction. Modulation of detuning also has a strong influence on the phase-relation and temporal relationship between rhythms and can set the direction of interaction.

Frequency detuning has several key advantages as a mechanism to define effective communication compared to changes in coupling. First, it is a potent mechanism to modulate synchronization. A shift of just a few Hz can make rhythms strongly coordinated or not. Second, it is a potent mechanism to tune the phase-relation (temporal relation). It modulates the time delay between the oscillations without any change in conduction delays. This allows flexible shaping of the precise temporal input pattern - traveling waves in spatial domain - to the down-stream neurons. For example, in the context of selective attention, input arriving earlier might be prioritized relative to later input (Tiesinga and Sejnowski, 2009, 2010). Or the temporal order of neural responses might be of importance for sensory object recognition (Gray and Singer, 1989; Eckhorn et al., 1990; Singer and Gray, 1995; Fries et al., 2001b).

### Concluding Remarks

Whether and to what extent frequency differences shape information flow and neural computation in the brain in a causal manner remains to be established. Demonstrating that a particular oscillation is necessary for a neural computation is difficult because experimentally removing an oscillation without affecting various other network properties is normally impossible, given that oscillations are usually emergent network phenomena (Buzsáki, 2006). Nevertheless, this review demonstrates that changes of a few Hz either between brain locations or a moment-to-moment basis, according to stimulus or cognitive conditions, is a property of neuronal oscillations in many frequency bands. Moreover, the precise oscillation frequency can have profound consequences in terms of synchronization properties (correlation, phase relations). Hence, we argue that manipulations of detuning represent a key experimental target to causally infer oscillatory properties without strongly affecting other network properties. Advanced multi-neuron electrophysiological and optical techniques (optogenetics, cellular voltage imaging) in future studies will provide exciting possibilities to measure and target frequency generation in neural oscillations and synchronization during cognition and behavior.

## Author Contributions

EL initially drafted the manuscript. All authors contributed to the final manuscript.

## Conflict of Interest

The authors declare that the research was conducted in the absence of any commercial or financial relationships that could be construed as a potential conflict of interest.

## Publisher’s Note

All claims expressed in this article are solely those of the authors and do not necessarily represent those of their affiliated organizations, or those of the publisher, the editors and the reviewers. Any product that may be evaluated in this article, or claim that may be made by its manufacturer, is not guaranteed or endorsed by the publisher.
